# Effects of a Superabsorbent Resin with Boron on Bacterial Diversity of Peat Substrate and Maize Straw

**DOI:** 10.1155/2018/6071085

**Published:** 2018-09-18

**Authors:** Yuxin Wang, Chaonan Wang, Yanan Zhao, Pingzhi Wang

**Affiliations:** College of Water Resources & Civil Engineering, China Agricultural University, Tsinghua East Road, Haidian District, Beijing 100083, China

## Abstract

As a chemical water-saving material, superabsorbent resin is often applied to improve soil physicochemical properties for the purpose of promoting crop growth. In this study, a new type of superabsorbent resin with boron (SARB) was used as a functional material mixed with peat substrate and maize straw in percentages (mass ratio) of 0.05%, 0.1%, 0.15%, and 0.2%, respectively, and high-throughput sequencing technology was used to test bacterial diversity, analyzing and exploring ecological safety of the superabsorbent resin with boron (SARB) in order to provide theoretical support for field applications. The research results show that the superabsorbent resin with boron (SARB) can promote bacterial community diversity in the maize straw. In ten treatments,* Proteobacteria* accounted for the absolute advantage of the bacterial population in the CT group and in the JG group. However, the superabsorbent resin with boron (SARB) synthesized in the laboratory cannot change the original structure of the bacterial community and has scarcely any toxic effect on the bacterial community in both peat substrate and maize straw, and, indeed, it has a strengthening effect on* Proteobacteria* and* Actinobacteria* and a weakening effect on* Acidobacteria* and* Firmicutes* to some extent.

## 1. Introduction

Microorganisms are the promoters of energy conversion and chemical elements cycling in soil [1]. The microbiological status in soil is not only one of the important factors of soil fertility evaluation, but also an important indicator of soil self-purification capability [2]. Microorganisms' changing characteristics are closely related to soil environmental quality, and therefore it is also the most direct and sensitive parts of the soil system affected by external disturbances. Current researches in the field of soil microbiology mainly include four aspects: species diversity, structural diversity, functional diversity, and genetic diversity [3]. Microbiological detection techniques which commonly used are plate separation culture techniques, DNA sequencing technology, molecular fingerprinting technology represented by DGGE, and high-throughput sequencing technologies. Compared with other technologies, high-throughput sequencing technology has the advantages of high flux and powerful analytical capabilities [4]. The high-throughput sequencing technology can simultaneously detect dominant species, rare species, and unknown species in the samples [5]. It has been widely used in the study of soil, food, intestine, wastewater, and other fields of research [6–9]. With the large-scale application of high-throughput sequencing technology in the ecological environment, the original nonculturable microorganisms in the soil also can provide abundant sequence information which may become the basis of studying such as microbial species in structure, function, and genetic diversity and so forth [10]. Relying on this technology, Oberholster [11] studied microbial communities in the rhizosphere of sorghum and sunflower grown in crop rotation through 16S rRNA-based amplicon sequencing and found dominant species of rhizosphere microorganisms in the rotation. These predominant species perform the same function at different growth stages of different crop rotations and rotation crops.

Superabsorbent resin (SAR) is a new type of polymer material with powerful absorbent and water-holding capacity. It is mainly divided into synthetic polymeric materials, natural polymer modified materials, and organic-inorganic composite materials [12]. SAR is also known as the fourth largest agricultural chemical production material after chemical fertilizers, pesticides, and plastic film [13]. The synthesis processes of SAR mainly include bulk polymerization, aqueous polymerization, reversed-phase suspension polymerization, inverse emulsion polymerization and radiation polymerization etc. Of these processes, radiation polymerization is a relatively advanced method for the preparation of SAR. It has the advantage of strong penetrating power, cleanness, high efficiency, and short reaction time [14]. The SAR act as the water reservoir, slowly releasing water in the soil. Another advantage related to the use of SAR in agriculture is that it can reduce death rate of plants and increase the output of crops [15]. In the experiment conducted by Montesano [16], adding SAR to perlite, a low water-holding capacity soilless substrate, 1% or 2% (w/w) of this kind of hydrogel, can increase the container capacity of water 28% and 48%, respectively, with no decrease of air capacity, which revealed absence of phytotoxicity of the hydrogel. Cultivation trials on cucumber (on soil) and sweet basil (in soilless conditions) showed overall enhancement of plants growth and quality when hydrogel was added to growing media. Cellulose modified superabsorbent resin has good salt resistance, easy adjustment of pH value, good anti-biodegradability, and wide source of raw materials, and obviously, it has important environmental significance and economic value.

In this study, one kind of superabsorbent resin with boron (SARB) was synthesized under laboratory conditions and mixed with peat substrate and maize straw, and, after 21 days, high-throughput sequencing technology was employed to test the effects of SARB on bacterial community. This research is aimed to reveal the differences in bacterial community structure and species composition in peat substrate and maize straw after the SARB were added in. Furthermore, the ecological safety of the SARB will be determined for the purpose of field application.

## 2. Materials and Methods

### 2.1. Materials and Treatments

The study was conducted in a greenhouse (temperature, 24–28.5°C) and peat substrate was mixed with vermiculite and perlite at the ratio of 2: 1: 1 in the laboratory. Maize straw come from farmland. It was crushed by the grinder and passed through a 100-mesh sieve.

Two groups including peat substrate (CT) and maize straw (JG) were set up separately and each group contain five treatments in which CK was used as the control (Table 1). Plastic pots (height, 16 cm; diameter, 12.5 cm) were individually filled with peat substrate and maize straw, and the amount of water added in each pot kept at 60% of saturation capacity of experimental materials. Trial samples from the ten treatments were collected by core samplers on the 21st day, and then the ecological safety of SARB was detected through analyzing the bacterial abundance and diversity.

### 2.2. DNA Isolation, PCR Amplification, and MiSeq Sequencing

Microbial DNA was extracted from samples by means of the Qiagen QIAamp Fast DNA Stool Mini Kit according to manufacturer's protocols. The final DNA concentration and purification were determined by NanoDrop 2000 UV-vis spectrophotometer, and DNA quality was checked by 1% agarose gel electrophoresis. The V3-V4 regions of the 16S rRNA gene which conserved the target sequences found in bacteria were amplified by the primers 338F (5'-ACTCCTACGGGAGGCAGCAG-3') and 806R (5'-GGACTACHVGGGTWTCTAAT-3'). The PCR conditions were as follows: 3 min of denaturation at 95°C, then 27 cycles of 30s at 95°C, 30s for annealing at 55°C, and 45s for elongation at 72°C and a final extension at 72°C for 10 min. PCR reactions were performed in a total volume of 20 *μ*L containing 4 *μ*L of 5 × FastPfu Buffer, 0.4 *μ*L of FastPfu Polymerase, 2 *μ*L of 2.5 mM dNTPs, 0.8 *μ*L of each primer (5 *μ*M), and 10 ng of template DNA. All PCR amplicons were isolated from 2% agarose gels and purified with a DNA gel extraction kit (Axygen Biosciences, Union City, CA, USA). The DNA concentration of each PCR product was determined by QuantiFluor™-ST fluorescent quantitative system (Promega, USA) before sequencing and mixed with the appropriate proportion according to sequencing requirements.

Purified amplicons were pooled in equimolar and paired-end sequenced (2 ×300) on an Illumina MiSeq platform (Illumina, San Diego, USA) in accordance with the standard protocols issued by Majorbio Bio-Pharm Technology Co. Ltd. (Shanghai, China).

### 2.3. Sequence Analysis

The obtained raw sequences were analyzed by Trimmomatic and merged by FLASH [17]. Then, the remaining sequences were clustered into operational taxonomic units (OTUs) at 3% difference implemented by the aid of UPARSE (version 7.1 http://drive5.com/uparse/), and chimeric sequences were identified and removed by means of UCHIME. The taxonomy of each 16S rRNA gene sequence was analyzed by the RDP Classifier algorithm (http://rdp.cme.msu.edu/) against the Silva (SSU123) 16S rRNA database with a confidence threshold of 70% [18]. A representative sequence from each OTU was selected for downstream analysis.

### 2.4. Statistical Analysis

Alpha-diversity analyses, including community richness indices(Chao1, ACE), community diversity indices (Shannon, Simpson), and a sequencing depth index (Good's coverage), were calculated with the assistance of Mothur software [19]. Bacterial taxonomic distributions of sample communities were visualized through R-Software package. Beta diversity measurements including microbiota trees were calculated as the reference described by Schloss [20], and principal coordinate analyses (PCoA) based on OTU compositions were conducted [21]. A Venn diagram was implemented through R-Software package to show unique and shared OTUs. Wilcoxon rank-sum test [22], which took into account both statistical significance and biological relevance, was conducted to identify OTUs differentially expressed in peat substrate and maize straw.

## 3. Results

### 3.1. Validation of the Sequencing Accuracy and Taxonomic Composition

In order to test the differences of different dosage of SARB on microbiota structure, trial samples collected from ten treatments were subjected to Illumina MiSeq sequencing of the bacterial V3-V4 region of the 16S rRNA gene [23] and 350909 high-quality sequence reads were obtained. In total, 861 OTUs were clustered after randomly resampling, ranging from 416 to 490 OTUs per sample, at a 3% dissimilarity level. The values of indices which indicate no significant difference in estimating OTU richness (Chao and ACE) and community diversity such as Shannon (4.97±0.1 versus 4.92±0.15, P = 0.68) and Simpson (0.02±0.002 versus 0.02±0.004, P =0.68) were acquired.

However, judging from the values of ACE index and Chao index, it indicates that the JG group has a higher degree of community abundance. Because the value of Shannon index from the JG group is smaller than that of the CT group, It indicates that the CT group has a higher variety of bacteria. The estimated sample coverage (Good's coverage) is more than 99.8%, which indicates that the accuracy of sequencing is reliable (Table 2).

Among the five treatments in the JG group, the value of Shannon index from the JG_CK is the smallest, indicating that the SARB can help to improve diversity of bacterial community in the maize straw. From the analysis of Sobs index, it can be known that the relationship of bacterial community richness between the five treatments is JG_T3>JG_T1>JG_CK>JG_T4>JG_T2. Among the five treatments in the CT group, the biggest value of Shannon index belongs to CT_CK, indicating that the SARB has a certain weakening effect on the bacterial diversity in the peat substrate. From the analysis of the Sobs index, the relationship of bacterial community richness between the five treatment is CT_T4>CT_CK> CT_T1>CT_T2>CT_T3 (Table 3).

**Figure 1:**
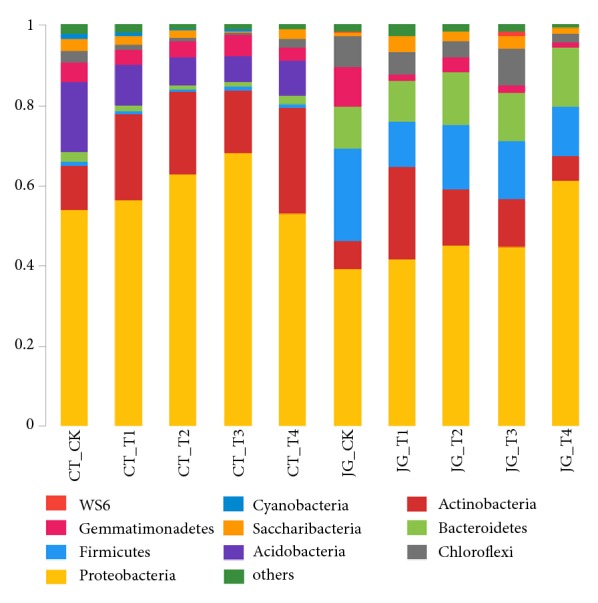
Community bar-plot analysis of relative abundance of microbiota at the phylum level.

All effective sequences were classified at the phylum level by aid of Mothur software with the default setting and 20 phyla were detected (Figure 1). Sequences which cannot be classified were assigned as no rank. In the ten treatments,* Proteobacteria *accounted for the absolute advantage in the bacterial community. In the CT group, the proportions of* Proteobacteria *were fluctuating with the changes of the mass ratio of the SARB. The proportion of* Proteobacteria *in the treatment of CT_T3 was the highest which run up to 68.04%. In the JG group, the proportion of* Proteobacteria *was rising with the increase of the mass ratio of the SARB. The proportion of* Proteobacteria *in the JG_T4 treatment was the highest which reached at 61.39%.

In the CT group,* Acidobacteria *and* Actinobacteria *were inferior to* Proteobacteria*. The proportions of* Actinobacteria *in the four treatments CT_T1, CT_T2, CT_T3, and CT_T4 were all higher than that of the CT_CK control, and, on the other hand, the proportions of* Acidobacteria *were totally lower than that of the CT_CK control. In the JG group,* Firmicutes*,* Actinobacteria*, and* Bacteroidetes *were inferior to* Proteobacteria*. The proportions of* Firmicutes *in the four treatments JG_T1, JG_T2, JG_T3, and JG_T4 were entirely lower than that of the JG_CK control, and, on the other hand, the proportions of* Bacteroidetes *were totally higher than that of the JG_CK control.

Obviously, the proportions of* Firmicutes *in the CT group were much lower than that of the JG group, and the proportions of* Acidobacteria *in the CT group was much higher than that of the JG group. This indicate that the SARB has a certain promoting effect on* Proteobacteria *and* Actinobacteria *and a certain weakening effect on* Acidobacteria *and* Firmicutes* (Figure 1).

### 3.2. Distribution of Bacterial Community

**Figure 2:**
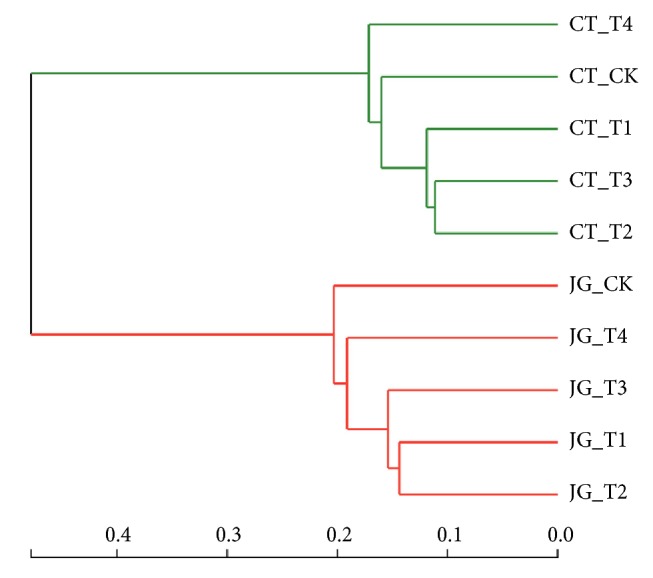
Clustering analysis of evolution of the microbiota in the JG group and CT group.

The relationship of microbiota in the 10 samples from the two groups was examined with aid of Bray–Curtis which displayed in the dendrogram (Figure 2) where each branch on the tree represented one sample of microbiota. Microbiota trees were established by aid of the UPGMA (unweighted pair group method with arithmetic mean) algorithm based on the Bray–Curtis distances generated by Mothur software. Obviously, the microbiota in the CT group clustered together on one branch and the microbiota in the JG group located on another branch. It indicates that the bacterial diversities of the five treatments in the CT group have similar feature, and the bacterial diversities of the five treatments in the JG group also have their own similar feature.

**Figure 3:**
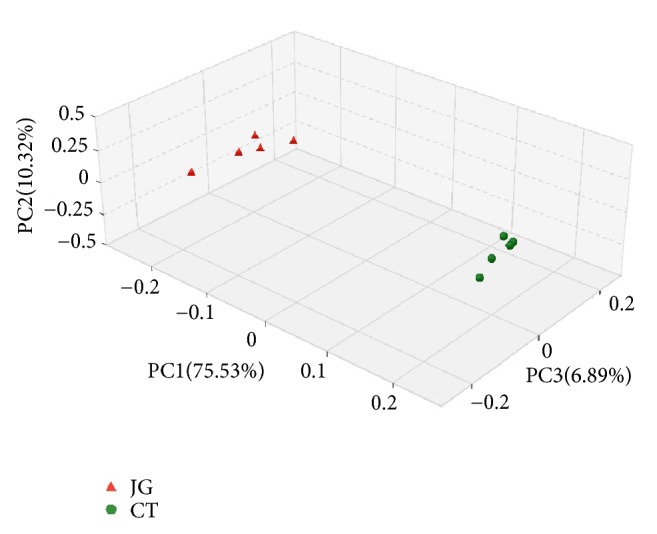
Principal coordinate analysis plot in the CT group and JG group.

Once closer analyses of microbiota differences between CT group and JG group are performed, the Weighted UniFrac PCoA plot based on OTU abundance can be mapped (Figure 3). Each point on the plot represents the microbiota of a sample in JG group (red triangle) or CT group (green circle), respectively. Similar to the cluster analysis, symbols of the same color are clustered together and have no intersection with symbols of another color, indicating differences in diversity. Principal coordinates analyses (PCoA) of sequencing data (the main principal component (PC) scores: PC1 = 75.53%, PC2 = 10.32% and PC3 = 6.89%) showed that corresponding to specific application environment of SARB, significantly different clusters of microbiota structure existed in JG group and CT group independently (Figure 3). This result is consistent with the dendrogram (Figure 2) where the microbiota in the CT group treated with SARB were found to possess almost the same number of OTUs.

### 3.3. Unique and Shared Bacterial Taxa

**Figure 4:**
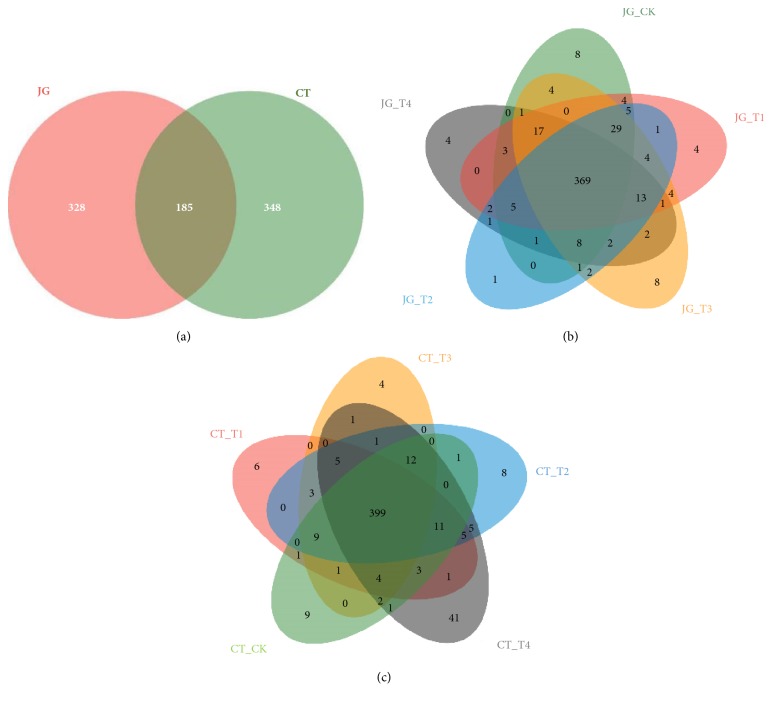
Unique and shared OTUs presented in the CT group and JG group.

Next, the shared and unique bacterial taxa in the two groups (CT group versus JG group) were examined by way of the sequencing data (Figure 4). The overlapping portion in Figure 4 indicates that the OTUs in it were shared by more than one sample packet, and the nonoverlapping portion indicates that these OTUs only belong to a specific sample packet, where the number represents the corresponding OTU numbers.

The shared 185 OTUs from the 10 samples are shown in Figure 4(a). Although both the CT group and the JG group possessed a large number of OTUs, there were significant differences on OTU categories in the two groups. There were 369 common OTUs in the group of JG, and the OTUs within this group held a high degree of reproducibility (Figure 4(b)) which indicates that, within a certain range of dosage, SARB has less effect on microbial community structure. There were 399 common OTUs in the CT group (Figure 4(c)) and the analysis results were similar to those of the JG group. However, the experiment results demonstrate that there existed great differences in the aspect of microbial community structure between the two groups.

**Figure 5:**
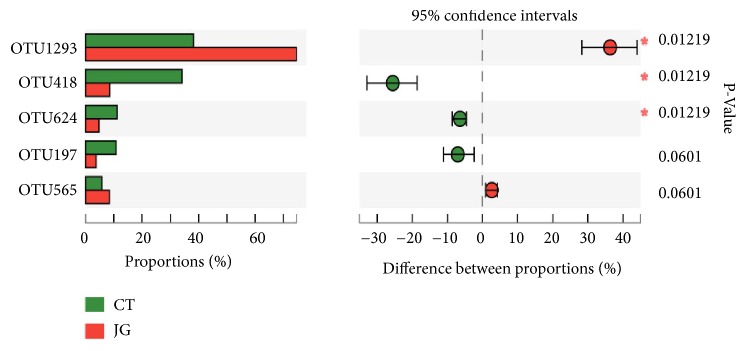
Differences of the top five abundances in the CT group and JG group at OTU level.

And then, the differential OTUs retaining the top five abundances in the CT group and JG group were identified by means of Wilcoxon rank-sum test (Figure 5). Statistical analysis showed that OTU1293 was affiliated with the genus* Escherichia-Shigella* and its abundance in the JG group was higher than that of the CT group. OTU418 and OTU624 were related to the genus* Ramlibacter* and the family* Caulobacteraceae*, respectively, and their abundance in the CT group is significantly higher than that of the JG group.

## 4. Discussion

Some previous studies on bacterial diversity in peat substrate were implemented based on culture-(in)dependent microflora analysis [24] or mini-clone libraries developed to determine the number of clones [25]. These reports uncovered some (un)culturable microbiota species and dominant microbiota flora, but the results cannot indicate the real microbiota structure. The development of high-throughput sequencing has allowed researchers to reveal the microbiota community at an unprecedented level compared with the traditional cultural-based method and PCR-DGGE way. Using high-throughput sequencing technology, we can more intuitively and in more detail understand the effects of different dosages of SARB on soil microbial community structure and species composition. Studies have shown that changes of environmental factors can significantly impact the diversity of microbial communities in soil [26, 27]. The application of SARB is not only conducive to solve the problem of soil boron deficiency, but also it can further promote the growth and development of crops.

## 5. Conclusion

The analysis of PCoA and Venn diagrams revealed that* Proteobacteria* constituted the core microbiota in all samples. The dosage of the SARB has different effects on bacterial diversity in CT and JG groups, which can promote bacterial community diversity in the maize straw. In terms of microbial growth, it means that the SARB has a strengthening effect on* Proteobacteria *and* Actinobacteria *and a weakening effect on* Acidobacteria *and* Firmicutes* to some extent.

The results indicate that the SARB synthesized in our laboratory has scarcely any toxic effect on the bacterial community in peat substrate and maize straw. It is apparently that the SARB synthesized has definitely ecological safety and a broad field application prospect.

## Figures and Tables

**Table 1:** Component of experimental treatments and grouping.

Method of application	Components
CT_CK	500g substrate
CT_T1	0.05% (w/w) SARB + 500g substrate
CT_T2	0.1% (w/w) SARB + 500g substrate
CT_T3	0.15% (w/w) SARB + 500g substrate
CT_T4	0.2% (w/w) SARB + 500g substrate
JG_CK	300g maize straw
JG_T1	0.05% (w/w) SARB + 300g maize straw
JG_T2	0.1% (w/w) SARB + 300g maize straw
JG_T3	0.15% (w/w) SARB + 300g maize straw
JG_T4	0.2% (w/w) SARB + 300g maize straw

**Table 2:** Richness and diversity indexes relative to CT group and JG group.

ID	Number of OTUs	Shannon	Simpson	ACE	Chao	Coverage
CT	458.4 ± 19.4	4.97 ± 0.1	0.02 ± 0.002	470.81 ± 22.04	477.06 ± 22.89	0.998 ± 0.0005
JG	456.2 ± 19.1	4.92 ± 0.15	0.02 ± 0.004	474.95 ± 14.60	479.92 ± 14.67	0.998 ± 0.0007
P-value	1	0.68	0.68	0.95	0.95	1

Data represents as mean ± standard deviation (SD). Statistical analyses were performed with Wilcoxon rank sum test between the two groups. The number of OTUs, richness estimator Chao and ACE, and diversity estimator Shannon and Simpson were calculated at 3% distance.

**Table 3:** Bacterial diversity indexes relative to the ten treatments.

Sample	Sobs	Shannon	Simpson	ACE	Chao	coverage
JG_CK	582	4.85	0.0204	635.69	646.20	0.9957
JG_T1	599	5.06	0.0168	635.97	632.50	0.9971
JG_T2	564	4.98	0.0148	603.22	605.89	0.9970
JG_T3	601	5.23	0.0104	637.39	652.00	0.9968
JG_T4	544	4.90	0.0152	623.72	623.84	0.9949
CT_CK	625	5.22	0.0123	665.03	685.64	0.9960
CT_T1	605	5.08	0.0140	646.65	656.33	0.9960
CT_T2	601	4.95	0.0172	662.53	666.11	0.9942
CT_T3	573	5.00	0.0162	622.50	623.52	0.9946
CT_T4	649	5.16	0.0142	711.16	716.90	0.9945

The community richness of bacteria is represented by Sobs index, Chao index, and ACE index, the larger values of which indicate the higher degree of community richness. Bacterial community diversity is represented by the Shannon Index and the Simpson Index. The larger value of Shannon index indicates that the diversity of the community is more abundant, and the larger value of Simpson index indicates lower diversity of the community.

## Data Availability

The raw sequence reads data used to support the findings of this study have been deposited in the NCBI repository (Accession: PRJNA474566).
